# Anti-Hyperglycemic Effect of Chebulagic Acid from the Fruits of *Terminalia chebula* Retz

**DOI:** 10.3390/ijms13056320

**Published:** 2012-05-22

**Authors:** Yi-Na Huang, Dong-Dong Zhao, Bo Gao, Kai Zhong, Rui-Xue Zhu, Yan Zhang, Wang-Jun Xie, Li-Rong Jia, Hong Gao

**Affiliations:** 1 Department of Public Health, Hua Xi Medicinal Center of Sichuan University, Chengdu 610041, China; E-Mails: dir0932@sina.com (Y.-N.H.); gaobo03@scu.edu.cn (B.G.); 2 Sichuan Academy of Medical Science & Sichuan Provincial People’s Hospital, Chengdu 610072, China; E-Mail: zhaodongd@gmail.com; 3 College of Light Industry, Textile and Food Engineering, Sichuan University, Chengdu 610065, China; E-Mails: eric211@163.com (K.Z.); zhu_ruixue@126.com (R.-X.Z.); yanzhang-scu@foxmail.com (Y.Z.); gaobingchuang@hotmail.com (W.-J.X.)

**Keywords:** *Terminalia chebula*, chebulagic acid, α-glucosidase inhibitor, anti-hyperglycemia

## Abstract

In the present study, we firstly compared rat intestinal α-glucosidase inhibitory activity by different ethanol-aqueous extractions from the dried fruits of *Terminalia chebula* Retz. The enzymatic assay showed that the 80% ethanol extract was more potent against maltase activity than both 50% and 100% ethanol extracts. By HPLC analysis, it was determined that the 80% ethanol extract had a higher content of chebulagic acid than each of 50% or 100% ethanol extract. Next, we investigated how efficiently chebulagic acid could inhibit sugar digestion by determining the glucose level on the apical side of the Caco-2 cell monolayer. The result showed that the maltose-hydrolysis activity was down-regulated by chebulagic acid, which proved to be a reversible inhibitor of maltase in Caco-2 cells. On the other hand, chebulagic acid showed a weak inhibition of sucrose-hydrolysis activity. Meanwhile, chebulagic acid did not have an obvious influence on intestinal glucose uptake and was not effective on glucose transporters. Further animal studies revealed that the oral administration of chebulagic acid (100 mg/kg body weight) significantly reduced postprandial blood glucose levels by 11.1% in maltose-loaded Sprague-Dawley (SD) rats compared with the control group, whereas the oral administration of chebulagic acid did not show a suppressive effect on postprandial hyperglycemia in sucrose- or glucose-loaded SD-rats. The results presented here suggest that chebulagic acid from *T. chebula* can be used to control blood glucose and manage type 2 diabetes, although clinical trials are needed.

## 1. Introduction

Diabetes mellitus (DM) is a common metabolic disorder characterized by hyperglycemia, which is the main cause of complications related with micro- and macro-vascular diseases. DM is one of the three leading causes of death worldwide and constitutes a major health problem [1]. Postprandial hyperglycemia results from abnormal insulin secretion by β-cells in response to a meal, impaired hepatic glucose production, and defective glucose uptake by peripheral insulin-sensitive tissues, particularly the skeletal muscles. Therefore, control of postprandial plasma levels is critical in treatment of not only diabetic patients but also individuals with impaired glucose tolerance [2]. Mammalian intestinal α-glucosidase (EC 3.2.1.20) is the key enzyme, which catalyzes the final step in the digestive process of carbohydrates. Hence, α-glucosidase inhibitors can reduce postprandial blood glucose levels and absorption of starch and disaccharides [3]. Plant-derived non-nutrients were recently found to play potent roles in modulating postprandial hyperglycemia. It has been reported that food and herbs are rich sources of α-glucosidase inhibitors [4–6].

The fruits of *Terminalia chebula* Retz (Combretaceae), also known as Xi-Qin-Ge in China, are a popular folk medicine for the treatment of various diseases, including digestive problems, diabetes, colic pain, chronic cough, sore throat and asthma. This plant has been studied for its biological activities, such as antioxidant [7,8], anticancer [9] and antibacterial activity [10]. Recently, *T. chebula* was reported to show an anti-diabetic effect [11–13]. A number of chemical compounds, including polyphenols [14], tannins [15] and triterpenoids [16], have been isolated from this plant species.

In the screening assay of α-glucosidase inhibitory activity for medicinal plants in China, we found that the methanol extract from the fruits of *T. chebula* showed a potent maltase inhibitory activity and the active compounds, chebulanin, chebulagic acid and chebulinic acid, were identified from this plant species [17]. As a continued study, the inhibitory mode of action of chebulagic acid on the rat intestinal maltase-glucoamylase complex was determined [18]. In the present study, our objective was to evaluate the intestinal α-glucosidase inhibitory effect by chebulagic acid from the fruits of *T. chebula in vitro* and *in vivo*. Therefore, we compared rat intestinal α-glucosidase inhibitory activity of different ethanol-aqueous extractions and detected chebulagic acid in these extracts by HPLC analysis. Meanwhile, we determined α-glucosidase inhibitory activity of chebulagic acid with the Caco-2 cell monolayer, together with evaluating the postprandial blood glucose lowering effect of chebulagic acid after sugar (maltose, sucrose or glucose) loading in Sprague-Dawley (SD) rats.

## 2. Results and Discussion

### 2.1. Isolation of Chebulagic Acid from *T. chebula* Fruits

**Figure 1:**
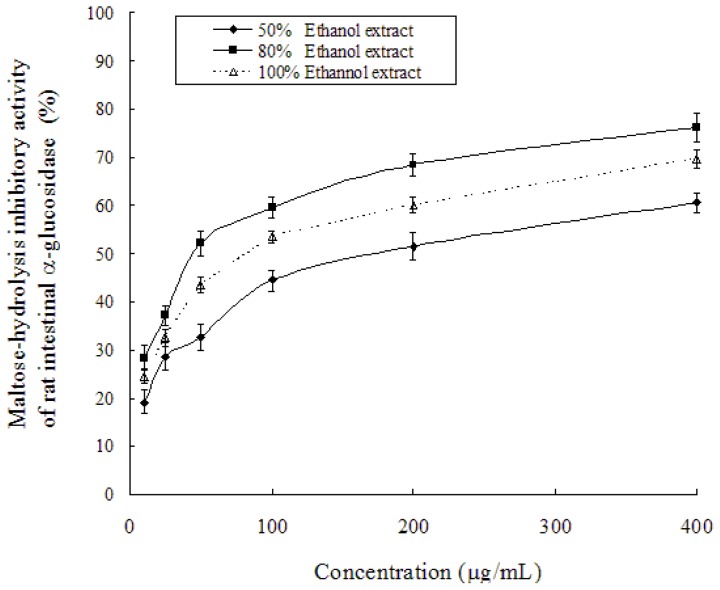
Maltose-hydrolysis inhibitory activity of rat intestinal α-glucosidase by 50–80% ethanol extracts from the fruits of *Terminalia chebula* Retz.

Extraction of phenolic compounds from *T. chebula* is generally carried out using various types of organic solvents such as 95% ethyl acetate, hot water, 70% methanol, and 95% ethanol [19]. Ethanolic extraction of plant bioactives has displayed a higher yield compared with the aqueous extract [20]. In the present study, we compared rat intestinal α-glucosidase inhibitory activity of different ethanol-aqueous extractions. Figure 1 shows the maltose-hydrolysis inhibitory activity of 50–100% ethanol extracts of *T. chebula* fruits. Each of these ethanol extracts significantly inhibited the maltase activity, and the enzymatic inhibition was dose-dependent. In the same assay condition, the IC_50_ value of chebulagic acid was determined to be 37 μg/mL (data not shown). On the other hand, the IC_50_ values of 50%, 80% and 100% ethanol extracts against maltase were determined to be 173.6 μg/mL, 51.7 μg/mL and 85.7 μg/mL, respectively. Hence, this result revealed that the 80% ethanol extract was more potent regarding its effect on maltase activity than both 50% and 100% ethanol extracts.

**Figure 2:**
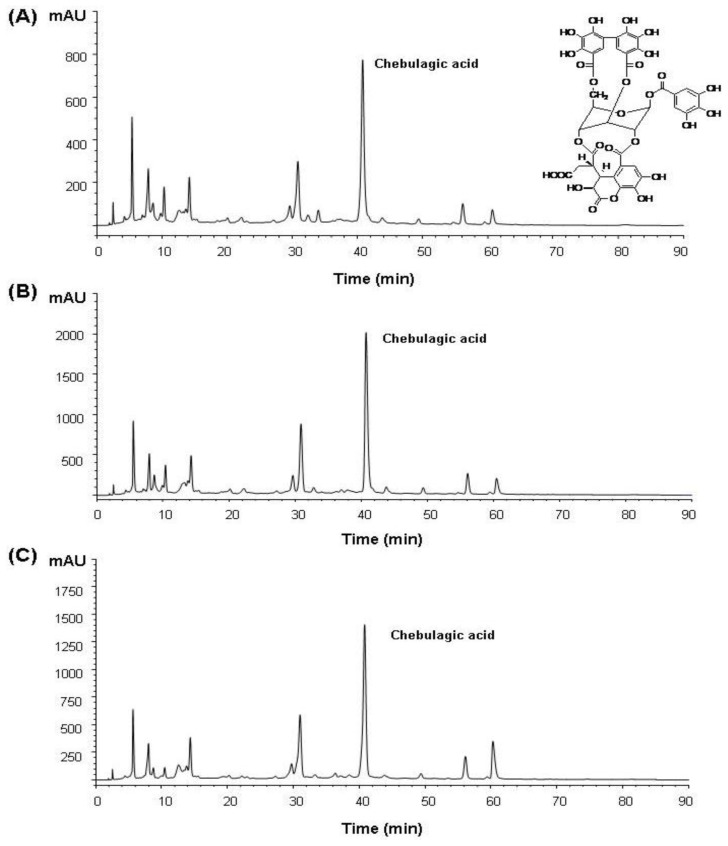
HPLC-DAD chromatograms of 50–100% ethanol extracts from the fruits of *Terminalia chebula* Retz. (**A**), 50% ethanol extract; (**B**), 80% ethanol extract; (**C**), 100% ethanol extract.

In order to ensure chebulagic acid in each ethanol extract, HPLC was used for further analysis. The compound at retention time 40.8 min was identified to be chebulagic acid, which was not found in the standard compound (Figure 2). Peak areas (mAU × Time (s)) of chebulagic acid identified in 50%, 80% and 100% ethanol extracts were determined to be 2.55 × 10^4^, 6.23 × 10^4^ and 4.61 × 10^4^, respectively. The 80% ethanol extract contained ~2.44- and ~1.35-times higher amounts of chebulagic acid than the 50% and 100% ethanol extracts. Therefore, chebulagic acid was prepared with our previous reported method [17] except that the dried fruit powders of *T. chebula* were extracted with 80% ethanol instead of 70% methanol, and the yield of chebulagic acid was about 7.3%. The chemical structure of chebulagic acid was identified with ^1^H-NMR and MS and the data were compared with data in the literature [17].

### 2.2. α-Glucosidase Inhibitory Activity in Caco-2 Cells

At late confluency and in culture, the human colon carcinoma cell line Caco-2 expresses the same morphological characteristics and most of the functional properties of terminally differentiated small-intestinal enterocytes, including the expression of proteins involved in the terminal digestion and uptake of sugars [21]. Caco-2 cells have been widely used as a culture model of human intestinal cells in studies to determine the α-glucosidase inhibitory effect by polyphenols [22–24]. In order to get the detailed information of chebulagic acid on mammalian intestinal α-glucosidase inhibitory activity, we investigated how efficiently chebulagic acid could inhibit sugar digestion by determining the content of liberated glucose on the apical side of the Caco-2 cell monolayer.

**Figure 3:**
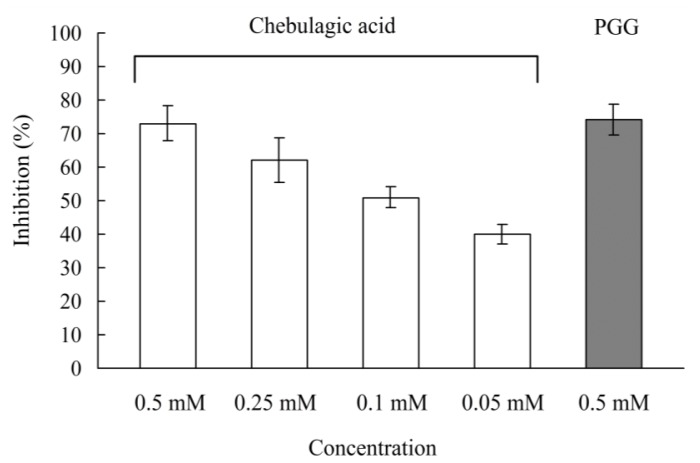
Results of the maltose hydrolysis assay with the Caco-2 monolayer in the presence of chebulagic acid (0.05–0.5 mM/well). PGG, 1,2,3,4,6-penta-*O*-galloyl-β-d-glucose (0.5 mM/well) as a positive control.

Firstly, the effect of chebulagic acid on Caco-2 cell viability was evaluated using an MTT assay. At growth doses of 0.05–0.5 mM/well of chebulagic acid, cell viabilities were in the ranges of 95–99% (data not shown). This result significantly indicated a non-cytotoxic property of chebulagic acid on Caco-2 cells. Then, Caco-2 cells were treated with the concentrations of 0.05–0.5 mM/well levels chebulagic acid at the apical sides and the liberated glucose was measured on the apical sides. After 2 h of incubation, α-glucosidse activity for the maltose hydrolysis was down-regulated, showing 73% inhibition at 0.5 mM/well, 62% inhibition at 0.25 mM/well, 51% inhibition at 0.1 mM/well, and 40% inhibition at 0.05 mM/well at the apical sides (Figure 3), respectively. PGG (1,2,3,4,6-penta-*O*-β-d-glucose) was used as the positive control, which showed 74% inhibition of the maltose hydrolysis of α-glucosidse activity in the Caco-2 monolayer at the concentration of 0.5 mM/well.

**Figure 4:**
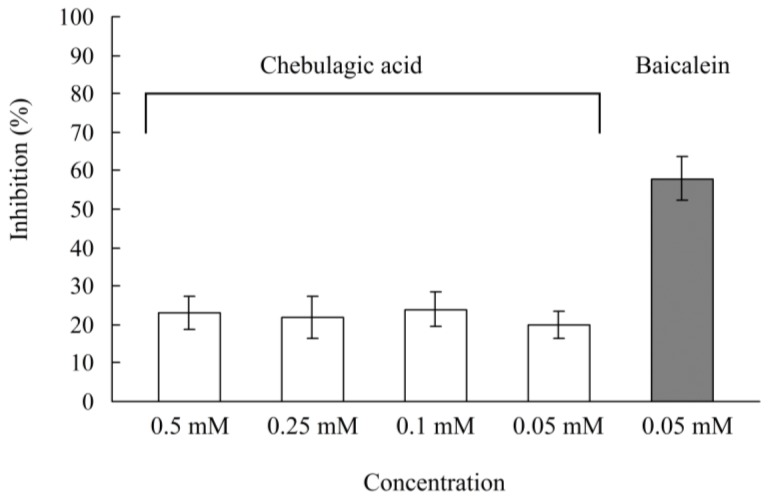
Results of the sucrose hydrolysis assay with the Caco-2 monolayer in the presence of chebulagic acid (0.05–0.5 mM/well). Baicalein, 5,6,7-trihydroxyflavone (0.05 mM/well) as a positive control.

On the other hand, chebulagic acid showed a weak inhibition against sucrose-hydrolysis activity of α-glucosidase with the Caco-2 monolayer. As shown in Figure 4, the inhibitory effect of chebulagic acid at the apical sides of the Caco-2 monolayer was determined, and the inhibitions were 20–24% at the concentrations of 0.05–0.5 mM/well levels. Baicalein (5,6,7-trihydroxyflavone), a potent sucrase inhibitor, showed 58% inhibition of the sucrose-hydrolysis activity of α-glucoside in the Caco-2 monolayer at the concentration of 0.05 mM/well. Our previous study reported that the inhibitory influence of chebulagic acid on the maltase-glucoamylase complex was more potent than on the sucrase-isomaltase complex [18]. In agreement with the rat enzyme assay, chebulagic acid affected the Caco-2 maltase reaction more than the Caco-2 sucrase reaction. Therefore, the anti-diabetic effect of chebulagic acid might be attributed to maltose-hydrolysis inhibitory activity of intestinal α-glucosidase, which can retard the digestion of carbohydrates resulting in mitigating postprandial hyperglycemic excursions.

**Figure 5:**
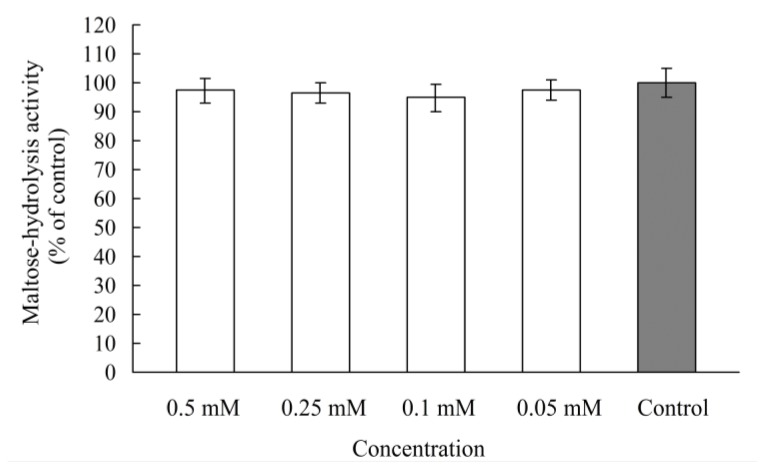
Maltose-hydrolysis activity in the Caco-2 monolayer pretreated with chebulagic acid (0.05–0.5 mM/well) for 20 min at 37 °C in 5% CO_2_ atmosphere.

In our previous study, analysis of a Lineweaver-Burk plot of rat intestinal maltase kinetics suggested that chebulagic acid shows a non-competitive inhibition, in which chebulagic acid and the maltose substrate seem to bind simultaneously to the enzyme [17]. In order to determine whether chebulagic acid is a reversible maltase inhibitor, Caco-2 cells were pretreated by chebulagic acid (0.05–0.5 mM/well levels) for 20 min at 37 °C in 5% CO_2_ atmosphere. Then it was examined whether the maltose-hydrolysis activity would be influenced or not in the Caco-2 monolayer. Compared with the control, the maltose-hydrolysis activities were in the ranges of 95–98% at the dose of 0.05–0.5 mM/well levels pretreatment with chebulagic acid (Figure 5). It is obvious that chebulagic acid is a reversible inhibitor of maltase.

**Figure 6:**
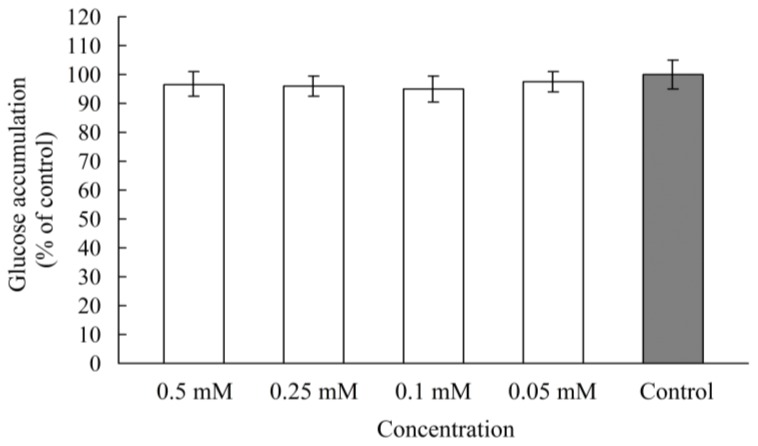
Glucose uptake in the Caco-2 monolayer in the presence of chebulagic acid (0.05–0.5 mM/well).

The liberated glucose from carbohydrate digestion is absorbed across the intestinal enterocytes via specific transporters, so glucose absorption is a potent target for better glycemia control after high-carbohydrate meals. Inhibition of glucose transporters would reduce the absorption into the small intestine and consequently suppress postprandial hyperglycemia [24]. A previous study reported that dietary polyphenols, such as (−)-epigallochatechingallate, (−)-epichatechingallate and (−)-epigallochatechin, are effective against glucose transporters and decrease glucose uptake by human intestinal Caco-2 cells [25]. In the present study, the effect of chebulagic acid on intestinal glucose uptake was investigated by using Caco-2 monolayers. As shown in Figure 6, compared with the control (glucose accumulation at the basal sides of the Caco-2 monolayer as 100% uptake), the glucose uptake rates were in the ranges of 94–98% in the presence of chebulagic acid (0.05–0.5 mM/well). This result suggests that chebulagic acid does not have an obvious influence on intestinal glucose uptake and has no effect on glucose transporters.

### 2.3. Effect of Chebulagic Acid on Blood Glucose Level in Sugar-Loaded SD-Rats

Our previous study showed that the oral administration of *T. chebula* fruit extract significantly reduced postprandial blood glucose levels in SD-rats after oral loads of maltose [26]. To clarify the active compound associated with the suppression of the rat blood glucose level, an anti-hyperglycemic effect of chebulagic acid in the extract was investigated.

**Figure 7:**
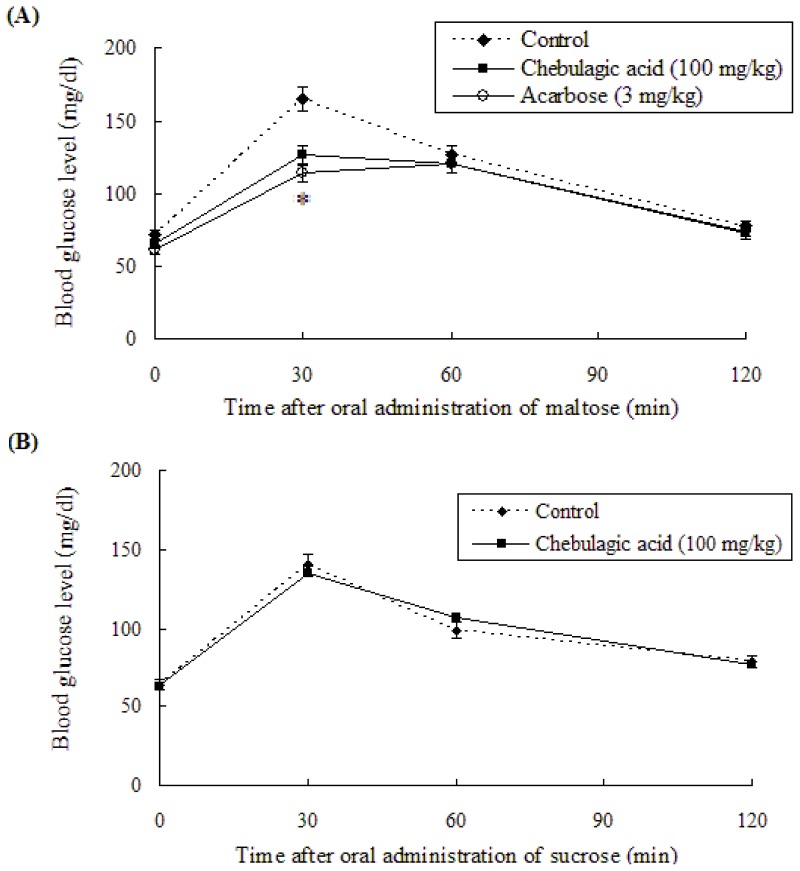

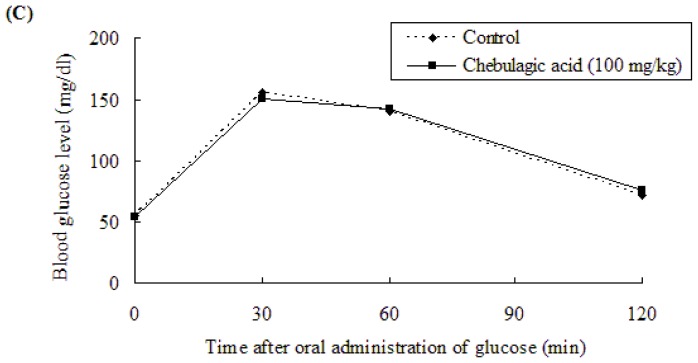
Anti-hyperglycemic effects of chebulagic acid on SD-rats. Fasted rats were given 2 g/kg of maltose (**A**), 2 g/kg of sucrose (**B**) and 2 g/kg of glucose (**C**), with or without 100 mg/kg of chebulagic acid (■) and vehicle (control: ◆). Acarbose (○) was used as a positive control for maltose-loaded SD-rats (**A**). Data are presented as the mean ± SD (*n* = 5). ^*^ *p* < 0.05 *vs.* control.

Figure 7 shows the anti-hyperglycemic effects of chebulagic acid in maltose (A)-, sucrose (B)-, and glucose (C)-loaded SD-rats. In the maltose-loading test, the blood glucose level in the chebulagic acid group was significantly lower than that in the control group (Figure 7A). The blood glucose level 30 min after administration was 126.7 ± 5.6 mg/dL in chebulagic acid (100 mg/kg, body weight) group, while in the control group the value was 165.2 ± 8.4 mg/dL. The raise in blood glucose level was reduced by 23.3% 30 min after gavage. The blood levels of chebulagic acid administrated SD-rats at 60 and 120 min were 120.2 ± 7.2 and 72.1 ± 6.9 mg/dL, respectively, while the values in the controls were 126.4 ± 4.8 and 77.0 ± 9.1 mg/dL, respectively. The area under the glycemic curve (AUC_0–120 min_) for chebulagic acid administrated SD-rats (207.8 ± 7.9 mg·h/dL of blood) showed a significant reduction of 11.1% compared with that of the control group (233.64 ± 6.6 mg·h/dL of blood). The acute intake of acarbose reduced the incremental AUC_0–120 min_ (199.6 ± 7.5 mg·h/dL of blood) in the SD-rats after maltose administration by 14.6%. Our animal study showed that the oral administration of chebulagic acid had a suppressive effect on hyperglycemia, which was similar to that seen with the administration of 3 mg/kg acarbose.

On the other hand, in the sucrose-loading test, the blood glucose level in the chebulagic acid (100 mg/kg, body weight) group was not significantly different from that of the control group (Figure 7B). As *in vitro* Caco-2 sucrase inhibitory activity of chebulagic acid was weak (Figure 4), there seemed to be no blood glucose level reduction in the sucrose-loaded SD-rats by chebulagic acid. The result was similar to the previous report in which methanol extracts of dried flowers of Ranawara (*Cassia auriculata*) with a potent maltase inhibition did not significantly reduce the postprandial blood glucose levels in sucrose-loaded rats, because Ranawara showed a weak sucrase inhibitory activity [27].

A glucose-loading test was also performed at a dose of 100 mg/kg. As shown in Figure 7C, the administration of chebulagic acid did not affect postprandial blood glucose levels in SD-rats after oral-loading glucose. According to the result obtained in glucose uptake by the Caco-2 monolayer (Figure 6), no blood glucose level reduction in glucose-loaded rats by chebulagic acid indicated that the significant hypoglycemic effect in maltose-loaded SD-rats resulted from intestinal maltase inhibition, not from intestinal glucose transporter inhibition.

## 3. Materials and Methods

### 3.1. Materials

The dried fruits of *T. chebula* were purchased from a local herbal market in Chengdu, China, and properly identified at the Department of Pharmacology, Hua Xi Medicinal Center of Sichuan University. The voucher specimen (No. 2011070202) is deposited in the department of Public Health, Hua Xi Medical Center of Sichuan University. Maltose was purchased from Merck Chemical Supplies. All other chemicals were purchased from Sigma-Aldrich Company unless otherwise stated. All other chemicals used including the solvents were of an analytical grade.

### 3.2. Preparation of Sample Extraction

The dried fruits of *T. chebula* were powdered in a blender. For extraction, every gram of the powered fruits was suspended in 20 mL of an extraction solvent: One hundred percent ethanol, 80% ethanol (ethanol/water = 8:2, v/v) or 50% ethanol (ethanol/water = 1:1, v/v). Extractions were carried out at room temperature for 36 h with continuous stirring and then treated with ultrasonication for 1 h. After filtration, the solvent from this extract was evaporated and the residues were lyophilized. The lyophilized powders were stored at −20 °C before use.

### 3.3. Determination of Chebulagic Acid in Each Extract

An amount of 10 mg of each of the above-mentioned extracts was dissolved in 1 mL of 70% methanol (a final concentration of 10 mg/mL), which was then passed through a HP020 filter (Advantec). Then 20 μL of this filtrate was injected directly for reverse-phase-HPLC analysis. The HPLC system (Agilent 1200 Series Purification System) consisted of an injector (G1328B), a column oven (25 °C), a pump (G1311A), a diode array detector (G1315D), and a reverse-phase column (Inertsil PREP-ODS-3, 4.6 × 250 mm, 5 μm, GL-Science). A linear gradient elution system, using solvent A and B (A = methanol; B = 0.1% formic acid in ultrapure water) according to the following profile: 0–10 min, 10–20% A, 90–80% B; 10–30 min, 20–30% A, 80–70% B; 30–50 min, 30–40% A, 70–60% B; 50–70 min, 40–50% A, 60–50% B; 70–90 min, 50–100% A, 50–0% B). The flow rate was maintained constant at 1.2 mL/min, and the phenolic compounds were monitored at 280 nm.

### 3.4. Isolation of Chebulagic Acid

Chebulagic acid was prepared with our previous reported method [17] except that the dried fruit powders of *T. chebula* were extracted with 80% ethanol instead of 70% methanol. The chemical structure of chebulagic acid was identified with ^1^H-NMR and MS and the data were compared with data in the literature [17].

### 3.5. Intestinal α-Glucosidase Inhibitory Activity

The α-glucosidase inhibitory activity was measured as described previously [17]. The crude enzyme solution prepared from rat intestinal acetone powder (Sigma-Aldrich) was used as the small intestinal α-glucosidase. The reaction mixture consisted of a crude enzyme solution (0.05 mL), a substrate (maltose, 3.5 mM, 0.35 mL) in 0.1 M potassium phosphate buffer (pH 6.3), and the test sample in 50% DMSO (0.1 mL). After incubation for 15 min at 37 °C, the reaction was stopped by adding 0.75 mL of 2 M Tris-HCl buffer (pH 7.0). The reaction mixture was passed through a short column of basic alumina, and the amount of liberated glucose was measured by the glucose oxidase method. The concentration of inhibitors required for inhibiting 50% of the α-glucosidase activity under the assay conditions was defined as the IC_50_ value.

### 3.6. Assay for the Caco-2 Cells Experiment

The intestinal epithelial cell line, Caco-2, derived from a human colon adenocarcinoma was used as an intestinal membrane model. Caco-2 cells used in this study were obtained from the National Chengdu Center for Safety Evaluation of Drugs. Cell viability was estimated by the MTT (3-(4,5-dimethylthiazol-2-yl)-2,5-diphenyltetrazolium bromide) assay. Briefly, Caco-2 cells were seeded in a 96 well plate at 1 × 10^5^ cells/mL. Twenty-four hours after plating, cells were treated with various concentrations of test sample and were incubated for an additional 2 h at 37 °C. After the cell culture medium was removed, the cells were incubated with 20 μL of MTT solutions (5 mg/mL) in 100 μL of medium at 37 °C for 4 h. The insoluble derivative formed by cellular dehydrogenase was solubilized with DMSO and absorbance was measured at 490 nm with a microplate reader.

Alpha-Glucosidase inhibitory activity in Caco-2 cell lines was performed with the previous method [28]. Briefly, Caco-2 cells were fed on polyethylene terephthalate membranes (Falcon, pore size: 0.4 μm, pore density 1.6 × 10^6^ pores/cm^2^, diameter: 23.1 mm) in a 6 well plate. After 16 days, the cell culture medium was removed and both the apical and basal chambers were washed 3 times with 2 mL of phosphate-buffered saline (PBS). The culture medium in the apical chamber was replaced with a reaction mixture containing chebulagic acid (0.05 mL) and a sugar solution (28 mM maltose or 28 mM sucrose) in PBS (0.95 mL) as a substrate. In the basal chamber 1 mL of PBS was added instead of the culture medium. After the Caco-2 enzyme reaction at 37 °C in 5% CO_2_ atmosphere for 2 h, 500 μL of each bathing solution on the apical side was transferred to pass through a short column of basic alumina, and the content of liberated glucose was determined by the glucose oxidase method.

The effect of chebulagic acid on α-glucosidase inhibitory activity was observed in Caco-2 cell lines. Briefly, Caco-2 cells (1.6 × 10^6^/mL) were fed onto a 12 well plate. After 16 days, the cell culture medium was removed and cells were treated with chebulagic acid in 1 mL of PBS. After 20 min incubation at 37 °C in 5% CO_2_ atmosphere, the treatment solution in each well was removed and the cells were washed twice with PBS. Then, the maltose-hydrolysis activity in the Caco-2 cells was determined as described.

The effect of chebulagic acid on glucose uptake was tested with Caco-2 cells lines in a 6 well plate. After 16 days, the cell culture medium was removed and both the apical and basal chambers were washed 3 times with 2 mL of phosphate-buffered saline (PBS). The culture medium in the apical chamber was replaced with a reaction mixture containing chebulagic acid (0.05 mL) and 28 mM glucose in PBS (0.95 mL). In the basal chamber 1 mL of PBS was added instead of the culture medium. After incubation at 37 °C in 5% CO_2_ atmosphere for 2 h, the glucose levels on the basal side were determined as described.

### 3.7. Animal Experimental

Six-week-old male Sprague-Dawley (SD) rats were purchased from the Laboratory Animal Center, Sichuan Academy of Medical Sciences. SD-rats were housed in an air-conditioned environment and with a 12 h light/dark cycle. The temperature was maintained at 22 ± 1 °C and the relative humidity was 55 ± 10%. The animals were provided with standard laboratory animal feed and water *ad libitum*. The SD-rats were fasted for 12 h and divided into groups according to body weight (200–250 g, *n* = 5). Each sample was dissolved in water and administrated by gavage. In the control group, 1 mL of 2 g/kg of a sugar (maltose, sucrose or glucose) solution was administrated to each rat. In the sample-treated group, SD-rats were given chebulagic acid (100 mg/kg body weight) or a positive control, acarbose (3 mg/kg body weight). Ten minutes after oral administration, the sugar-loading test was done. Blood samples were collected from the tips of the rats’ tails at 30, 60, 90 and 120 min after each sugar loading. The blood glucose levels were measured immediately with a commercial glucometer and test-strips (GT-1640, Kyoto, Japan).

All experiments were carried out in accordance with the ethical guidelines of the Sichuan Province Experimental Animal Management Committee and were in complete compliance with the National Institutes of Health Guide for the Care and Use of Laboratory Animals.

### 3.8. Statistical Analysis

Data were reported as mean ± standard deviation of mean. Statistical analyses were performed with Student’s *t*-test using the SPSS program (version 12.0 for Windows, SPSS Inc., Chicago, IL, USA). Values were considered to differ significantly if the *p* value was less than 0.05.

## 4. Conclusions

Mammalian intestinal α-glucosidase inhibitors can reduce postprandial plasma glucose levels and absorption of starch and disaccharides, and play potent roles in modulating postprandial hyperglycemia. In a previous study, we reported that the methanol extract from the fruits of *T. chebula* showed a potent maltase inhibitory activity and the active compounds, chebulanin, chebulagic acid and chebulinic acid, were identified from this plant. In the present study, we compared rat intestinal α-glucosidase inhibitory activities of different ethanol-aqueous extractions from the dried fruits of *T. chebula*. The enzymatic assay showed that the 80% ethanol extract had a more potent effect on maltase activity than both 50% and 80% ethanol extracts. HPLC analysis revealed that the 80% ethanol extract contained a higher content of chebulagic acid than either the 50% or 100% ethanol extract. In a Caco-2 cell model, α-glucosidase activity for maltose hydrolysis was down-regulated by chebulagic acid, which is a reversible inhibitor of maltase. On the other hand, chebulagic acid showed a weak inhibition of sucrose-hydrolysis activity and did not affect intestinal glucose uptake by Caco-2 cells. Furthermore, chebulagic acid significantly reduced postprandial blood glucose level in maltose-loaded SD-rats. So, it was suggested that chebulagic acid from *T. chebula* may be useful for suppressing postprandial hyperglycemia as a potent anti-diabetic agent, although clinical trials are needed.
